# Individual separation of surface, bulk and Begrenzungs effect components in the surface electron energy spectra

**DOI:** 10.1038/s41598-021-85429-6

**Published:** 2021-03-15

**Authors:** Lihao Yang, Bo Da, Károly Tőkési, Z. J. Ding

**Affiliations:** 1 Hefei National Laboratory for Physical Sciences at Microscale and Department of Physics, University of Science and Technology of China, Hefei, 230026 Anhui People’s Republic of China; 2 Research and Services Division of Materials Data and Integrated System, National Institute for Materials Science, 1-1 Namiki, Tsukuba, Ibaraki 305-0044 Japan; 3 Research Center for Advanced Measurement and Characterization, National Institute for Materials Science, 1-2-1 Sengen, Tsukuba, Ibaraki 305-0047 Japan; 4 Institute for Nuclear Research (ATOMKI), Debrecen, Hungary

**Keywords:** Computational methods, Surfaces, interfaces and thin films

## Abstract

We present the first theoretical recipe for the clear and individual separation of surface, bulk and Begrenzungs effect components in surface electron energy spectra. The procedure ends up with the spectral contributions originated from surface and bulk-Begrenzungs excitations by using a simple method for dealing with the mixed scatterings. As an example, the model is applied to the reflection electron energy loss spectroscopy spectrum of Si. The electron spectroscopy techniques can directly use the present calculation schema to identify the origin of the electron signals from a sample. Our model provides the possibility for the detailed and accurate quantitative analysis of REELS spectra.

## Introduction

As early as 1957, Ritchie theoretically predicted the excitation of surface plasmons of thin films by fast electrons. Two years later, following the theoretical prediction, Powell and Swan^1,2^ discovered this kind of excitation experimentally in the spectra of two free-electron-like materials, i.e. aluminum and magnesium. Since the first observation of surface excitations especially hot topic of interest is to develop a method or technique to separate the surface and bulk properties as observed by electron spectroscopy. We note that, in Ritchie’s pioneering work^3^, the surface effect was already divided into two parts: one of them is the additional surface modes of the polarization field in the vicinity of the surface, which have an excitation energy of about ${{\omega_{b} } \mathord{\left/ {\vphantom {{\omega_{b} } {\sqrt 2 }}} \right. \kern-\nulldelimiterspace} {\sqrt 2 }}$ where $\omega_{b}$ is the bulk-plasmon excitation energy, and the second one is the coupling between surface modes and bulk modes near a boundary, which results in a reduction of the intensity of bulk excitations. Such a decrease effect on the bulk excitation is known as Begrenzungs effect. The surface excitation together with the Begrenzungs effect forms the surface effect. By using the secondary-electron electron-energy-loss coincidence spectroscopy, a strong reduction of bulk mode in the surface scattering zone has been observed^4^. The low-loss electron energy loss spectra for Ti_3_C_2_*T*_2_ (*T* = OH or F) stacks of various thicknesses have been measured and it has been found that the intensity of bulk plasmon is significantly reduced as the Ti_3_C_2_*T*_2_ stack thickness is decreased^5^. The plasmon energy of a 2-nm GaN quantum well was larger than that of a relaxed GaN^6^. Those phenomena are considered to be due to the influence of the Begrenzungs effect. However, there is a lack of quantitative analysis methods for dealing with the Begrenzungs effect.

Energy loss of electrons near surfaces raises several interesting problems, among them is the separation of surface and bulk effects. In the standard electron spectroscopy techniques, it is not possible to resolve the clear, distortion-free separation of surface properties from the bulk one. This is due to the fact that electrons always penetrate into the material and move either deep inside the bulk or move near the surface region. The probability of the energy loss can be determined by the dielectric response function, $\varepsilon \left( {q,\omega } \right)$, which is a function of the frequency *ω* and the wavenumber $q$ of the electromagnetic disturbance. For the accurate theoretical modeling of the electron spectra, the surface effects and the multiple electron scattering in the inelastic interaction must be treated with special care. This special care is especially important at low incident energies and at grazing scattering geometries, where surface effects dominate.

Significant improvements in describing the surface excitations^7–20^ have been made in the last decade. Tougaard and Kraaer investigated the inelastic cross sections of several elemental materials using the reflected electron energy loss spectroscopy (REELS). They found that the accurate description of the surface excitation, which is enhanced at low incident energies, is very important in the quantitative analysis of REELS spectra^7^. The early theoretical approach employed a simple two-layer model to interpret the measured backscattered electron spectra^8,9^. The top layer with the thickness of several atomic monolayers is characterized with the surface energy loss function (ELF) and the bottom one with the bulk ELF. In some other previous works^10–12^, the surface and bulk excitations are considered as two independent events and the corresponding probabilities can be linearly superimposed in a dielectric functional formulation, thus, described by the surface and bulk ELFs, respectively. However, both these models are not so accurate, due to the reason that the surface effect in these two models is isotropic and will not occur in the vacuum while in a real sample it is depth-dependent and can also occur in the vacuum^13,14^. Based on a quantum mechanical approach, Ding^13–15^ has derived a formalism of position- and velocity-dependent electron inelastic scattering cross section near the surface region via a complex self-energy formula. This quantum mechanical model of the inelastic scattering was applied in the simulation of REELS spectrum for ideal flat Au, Si^16^, and Ag^17–19^ surface and rough Al surface^21,22^. However, we note here that this sophisticated quantum mechanical model is less computationally efficient compared with a semi-classical model^20^. It has been verified that the depth-dependent differential inverse inelastic mean free path (DIIMFP) produced by the quantum model and the semi-classical model is quite similar and the difference between the REELS spectra simulated by these two models is practically invisible^23^. Therefore, nowadays the semi-classical model, which effectiveness has been verified by many previous works^24–31^, is more frequently widespread. On the basis of the semi-classical dielectric response theory, a theoretical model for the DIIMFP for incident and escaping electrons in a layered structure sample has been developed^32^. By using this layered structure DIIMFP, the simulation of REELS spectrum for carbon contaminated SrTiO_3_ surface^33^ and Fe/Si overlayer sample^34^ have been performed.

Although we have in our hands good models to describe the surface effect, they are still not able to separate clearly the spectral components and do a further detailed quantitative analysis. A deconvolution method has been developed by Tougaard and Chorkendorff^35^ to extract the DIIMFP from REELS spectra. Such a deconvolution method has been applied to Al^35^ and Si^7^. The resulted DIIMFPs of Al and Si have negative values, which are non-physical, around $\omega_{b} + \omega_{s}$, where $\omega_{b}$ and $\omega_{s}$ are the bulk- and surface-plasmon excitation energy, respectively. This is due to that the influence of both the angular distribution of elastic scatterings and the surface effect are omitted. Their method has been improved by considering the surface effect^36^. A trial-and-error procedure was employed to find the best fitting ELF which can be used to calculate the DIIMFP in the best agreement with the DIIMFP extracted from experimental REELS^37^. However, there are still large deviations between the calculated and experimentally extracted DIIMFPs in the energy loss range up to $\omega_{b} + \omega_{s}$. Werner^38^ hypothesized that the bulk excitation and surface effect are uncorrelated and REELS spectrum can be expressed via a convolution of various excitations with the elastic peak. Then the energy loss distribution of single surface effect and single bulk excitation, which are named as the differential surface excitation probability (DSEP) and DIIMFP, can be extracted from the experimental REELS spectra based on a deconvolution approach. Based on the obtained DSEP and DIIMFP, they can revisit the REELS spectra and perform the quantitative analysis^39–41^. However, the generation mechanism of REELS spectra is very complex; it is made of elastic scattering, inelastic scattering, surface effect, multiple scattering effect as well as influenced by experimental condition. Therefore, the REELS spectra are hard to be expressed accurately by convolution formulation. Two peaks at 12 eV and 34 eV appear in retrieved bulk excitation DIIMFP of Si from experimental REELS spectra; this fact indicates that such a retrieved DIIMFP contains partial surface excitation (12 eV) and multiple scattering effect (34 eV). This is due to that the multiple scattering effect and the surface effect cannot be well deducted by using the deconvolution method.

On the other hand, Monte Carlo (MC) simulation method is a powerful tool for the simulation of electron-solid and electron-surface interaction. It can deal with the multiple scattering effect more accurately, and can be used to obtain both the electron energy spectra^29^ and secondary electron yields^42–44^ which are in good agreement with the experimental results. The quantitative analysis of REELS spectra can be done based on a MC simulation method^24,30^. Both the current deconvolution scheme and the MC simulations have a disadvantage, i.e., there is no more subdivision of the surface effect. The quantitative analysis of individual Begrenzungs effect and surface excitation cannot be performed based on the existing methods.

In this work, we present a recipe for individual separation of surface, bulk, and Begrenzungs effect components in the surface electron energy spectra. Our theoretical recipe is based on the evaluation of the depth-dependent DIIMFP. As an example, the present recipe is applied to the analysis of REELS spectra of Si at the primary energy of 5 keV.

## Results

**Figure 1:**
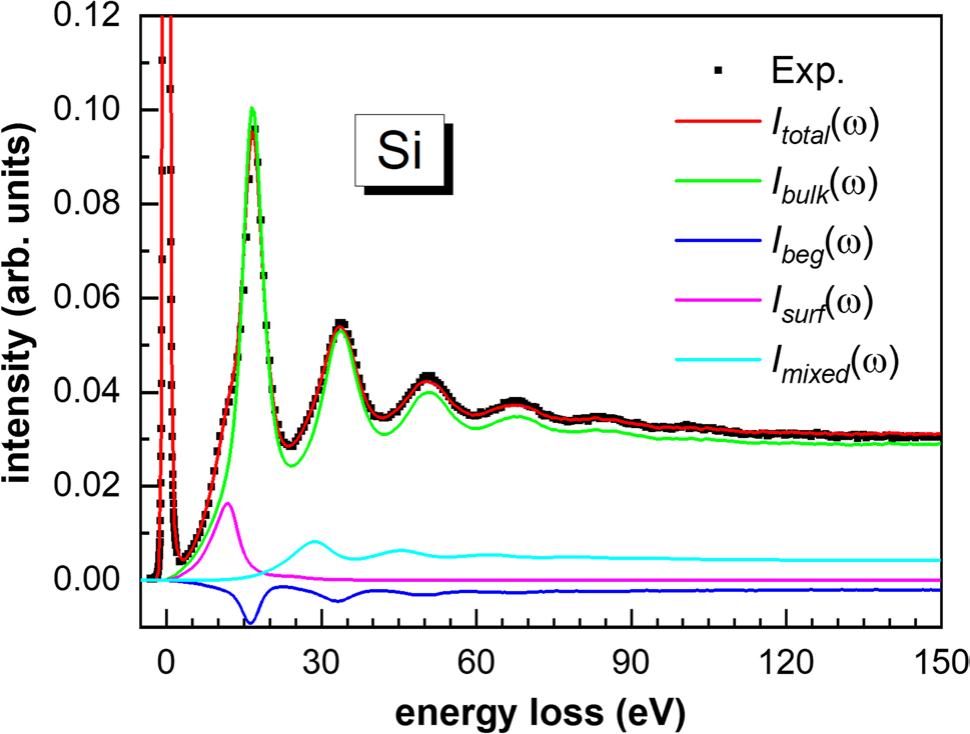
The experimental and simulated REELS spectra with the partial spectral components for Si at primary energy of 5 keV. Dotted line: experiments; red line: total simulated REELS spectra, green line: bulk excitation component, blue line: surface excitation component, magenta line: Begrenzungs effect component, cyan line: mixed term component.

**Figure 2:**
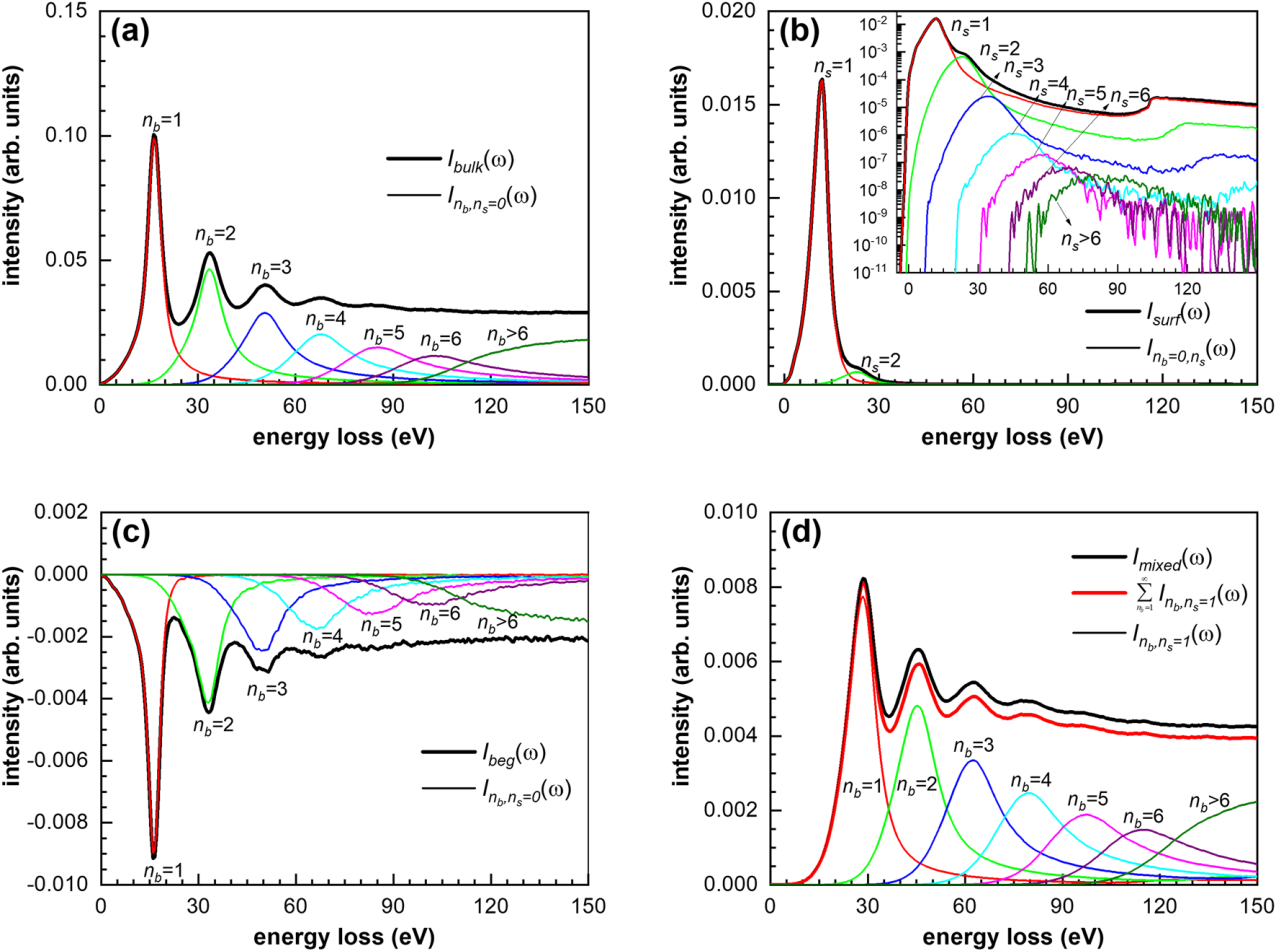
Multiple scattering terms of different components in the simulated REELS spectrum for Si at primary energy of 5 keV: (**a**) bulk excitation component; (**b**) surface excitation component; (**c**) Begrenzungs effect component; (**d**) mixed component.

Figure 1 shows the experimental and simulated total REELS spectra with the partial spectral components, as bulk, surface and mixed excitations and Begrenzungs effect components for Si at primary energy of 5 keV. The agreement between the total simulated REELS spectrum and the experiments is excellent. For each detected electron, the present recipe can trace the number of inelastic scatterings and the specific type for each single inelastic scattering. Therefore, it is straightforward the separation of the multiple scattering term for each component. Figure 2 shows the multiple scattering terms of different simulated components in the REELS spectrum for Si at 5 keV.

**Figure 3:**
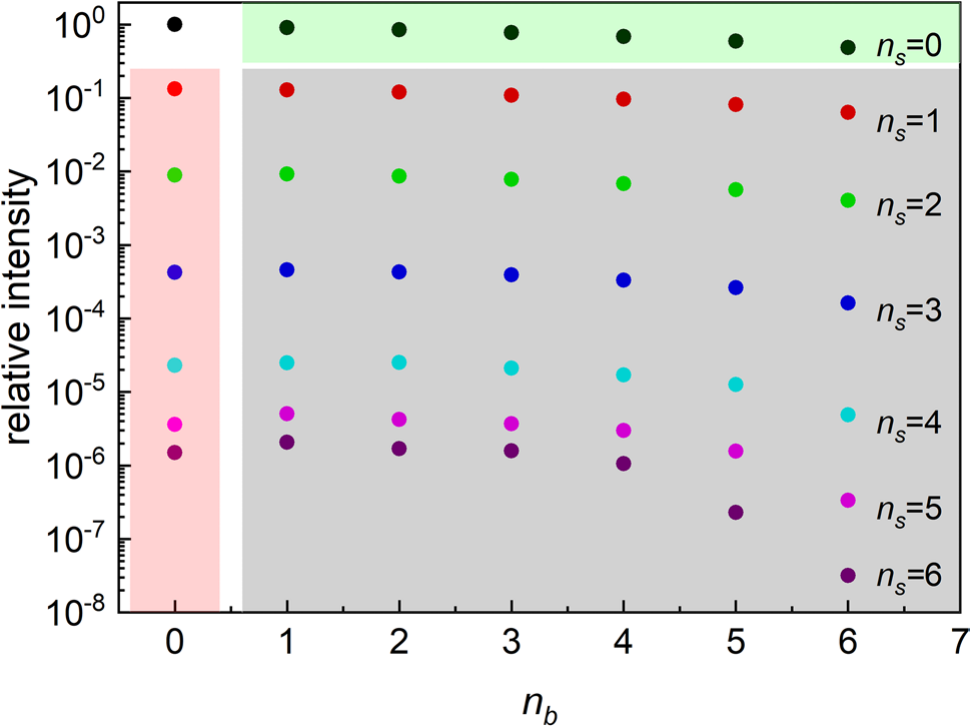
Comparison of the relative intensity of $I_{{n_{b} ,n_{s} }} \left( \omega \right)$ of REELS spectrum of Si at the energy of 5000 eV. Light green area: bulk-Begrenzungs component; light red area: surface excitation component; gray area: mixed term component.

According to Fig. 2 the signature of the multiple electron scatterings can be well characterized with separate peaks, where each peak can be assigned with an order of the multiple scattering. At higher electron energies, the single scattering for surface excitation dominates (Fig. 2b). The intensity of electrons which suffer no bulk excitation and more than two surface excitations is much stronger than intensity of electrons which suffer no bulk excitation only one surface excitation. In the mixed contribution we highlight the contribution dedicated to the single surface excitation, where again we can separate and well define peaks (Fig. 2d). In the absolute yield the bulk excitation is the largest and the yield of the mixed contribution is the smallest. Figure 3 shows a comparison of the relative yields for various orders of excitation components. The green area corresponds to the bulk excitation, the pink area corresponds to the surface excitation and the gray area corresponds to the mixed scattering component. Due to the localization of the surface effect, the intensity of $I_{{n_{b} ,n_{s} }} \left( \omega \right)$ term decreases rapidly with the increasing of the number of surface excitation $n_{s}$ as shown in Fig. 3.

**Figure 4:**
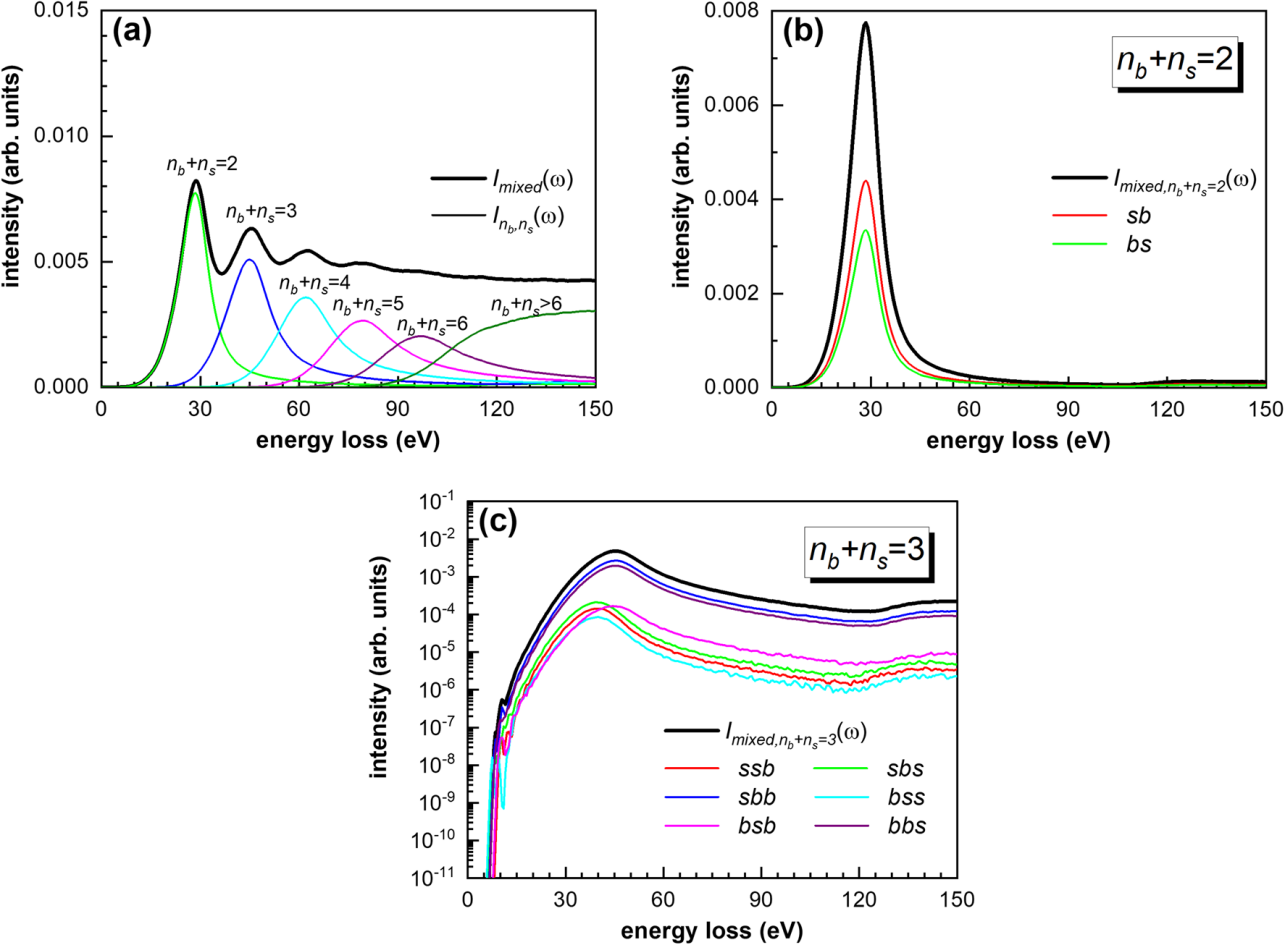
Spectral component of the mixed term: (**a**) total spectra; (**b**) partial component when $n_{b} + n_{s} = 2$; (**c**) $n_{b} + n_{s} = 3$ of the mixed scattering.

For the clear separation between the bulk and surface excitations, we need to analyze further the mixed scattering component. Figure 4 shows the total spectral component of the mixed term with two partial distributions when the number of inelastic collisions is 2 or 3. Here, we introduce the shorthand *b* and *s* to denote the bulk and surface scatterings, respectively. In this notation, the so-called bulk excitation due to the electron inelastic collision in the bulk is denoted by *b*, while the surface excitation due to the electron inelastic scattering in the surface region is denoted by *s*. Longer sequences can be referred to as, for example, *bs*, *sb*, or even more longer sequences like, *bss*, *sbs*, *ssb*. Moreover, the order from left to right of each symbol denotes the order of different collisions. In this notation, there are two types of collisions in the so-called double mixed collision. The first one when the first collision is in the bulk and the second collision is in the surface before the electron escape from the sample (*bs*). The second case when the first collision is in the surface and the second collision is in the bulk before the electron escape from the sample (*sb*). Using our MC simulation, we can directly calculate the corresponding contributions of the mixed terms. With increasing the number of collision, the number of different collision sequences increases drastically. In the case of 3 collisions, the number of cases is 6 (Fig. 4c). The intensities of *bs*, *bss*, and *bbs* are slightly lower than that of *sb*, *ssb*, and *sbb*, respectively. This behavior can be interpreted taken into account the different excitation probabilities when an electron passes through the surface region either from the vacuum to the sample or from the sample to the vacuum, i.e. $v_{ \bot } < 0$ or $v_{ \bot } > 0$ in Eqs. (2–4). Obviously, the surface excitation mainly occurs when electrons move from the sample to the vacuum (from the vacuum to the sample) in *bs*, *bss*, and *bbs* (*sb*, *ssb*, and *sbb*). The intensity of *bsb* is much lower than that of *sbb* and *bbs*, which clearly shows that the final collision order of an electron depends on the trajectory due to the depth-dependence of surface effect. The collision order of electron can be counted in detail mainly because the MC method traces the whole process of electron transport from entering to the sample to absorption or emission from the surface. This is an important advantage of the MC simulation method in the application quantitative analysis of surface electron energy spectra compared to the convolution method^38,45^.

## Discussions

**Figure 5:**
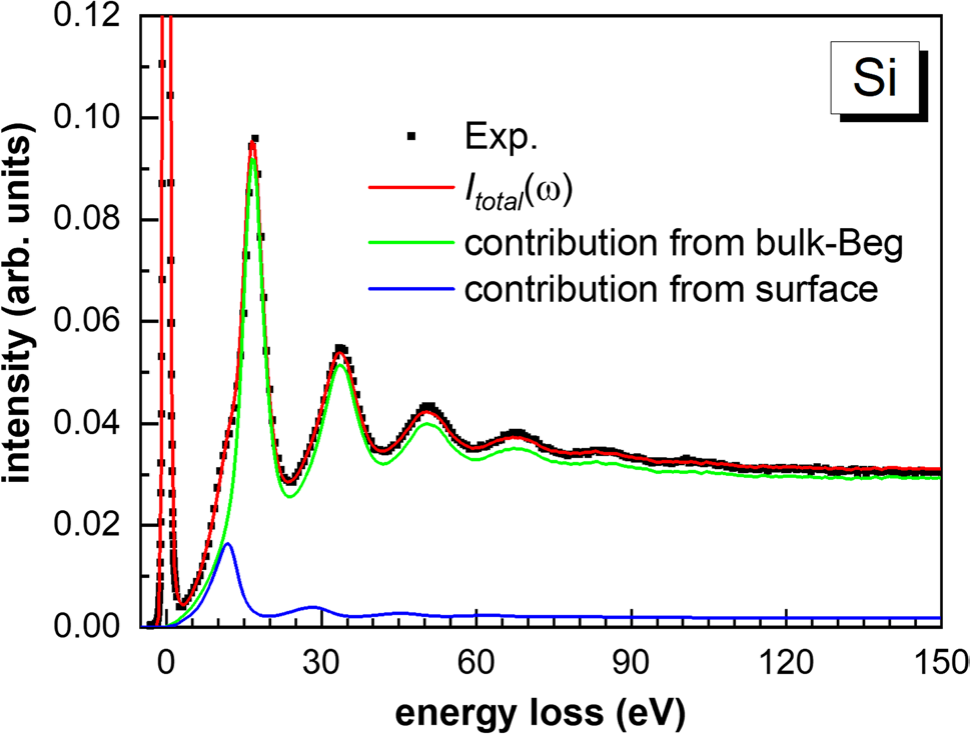
REELS spectral components originated from surface and bulk-Begrenzungs for Si at incident energy of 5 keV. black dots: measured data, red line: simulated REELS spectra, green line: contribution from the bulk including the Begrenzungs effect, blue line: contribution from the surface.

We note that there are two kinds of inelastic scatterings in each single collision when an electron passes through the surface of a sample, i.e. bulk and surface excitations. In order to classify and divide into two parts of a REELS spectra as only surface or bulk-Begrenzungs contribution, as the classification of each single collision, we need to deal with the mixed scatterings. One simple way is to classify the mixed scatterings regarding to if the last collision is surface or bulk before the electron escapes from the sample. Applying this scenario, the individual and separate surface and bulk excitation can be calculated. Figure 5 shows the mixing free individual separation of surface, bulk contribution for Si at incident energy of 5 keV.

In summary, a new theoretical recipe for the clear and individual separation of surface, bulk and Begrenzungs effect components in the electron spectra without any mixing between the components was shown. Our model is based on the evaluation of the depth-dependent differential inverse inelastic mean free path. By using this method, one can analyze the contribution from different components in a REELS spectrum in detail. The quantitative analysis of REELS spectrum of Si at the primary energy of 5 keV has been performed. Our work proves that single scattering for surface excitation dominates for the REELS spectrum of Si, due to the localization of the surface effect. The present analysis clearly shows that the final collision order of an electron depends on the trajectory due to the depth dependence of surface effect. This work extends the quantitative analysis method of REELS spectra into the more detailed and accurate realm.

## Methods

**Figure 6:**
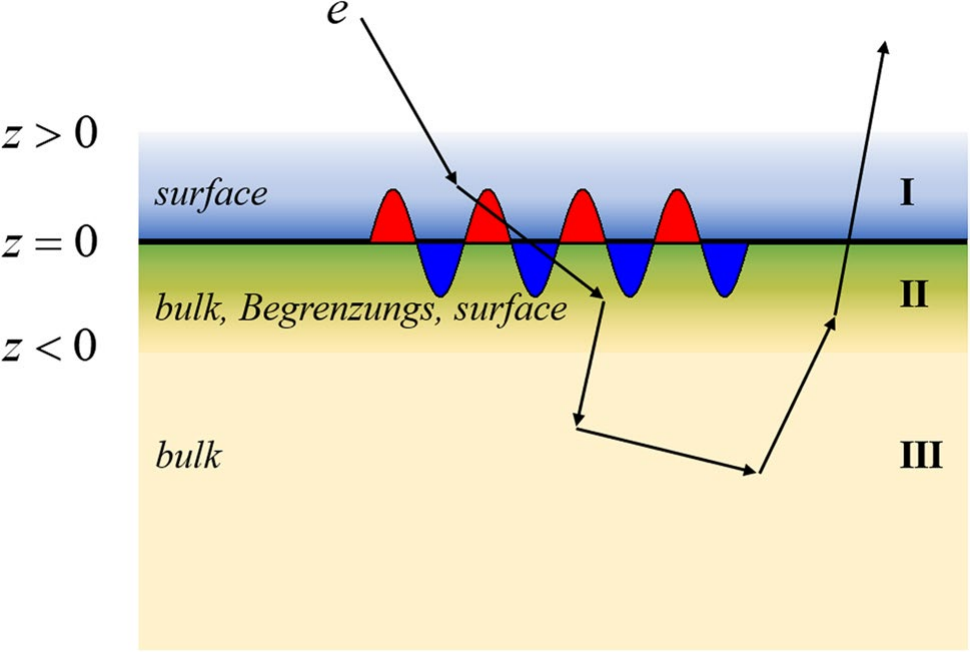
Schematic diagram of the hypothetic sample with typical electron trajectory.

The solid medium is considered to occupy a semi-infinite space with the surface boundary defined at $z = 0$. A sketch of the considered geometry for the problem by indicating the vacuum ($z > 0$) and solid ($z < 0$) regions is shown in Fig. 6. When an electron passes through a solid surface, elastic scattering occurs only inside the solid, while there are three situations to be considered for the inelastic scattering process. First, the electron is near the surface region of the vacuum (region I), where only surface excitation occurs. Second, the electron is near the surface region of the solid (region II), where the bulk excitation, Begrenzungs effect and the surface excitation jointly contribute to the inelastic scattering process. Third, the electron is in the interior region of the solid (region III), where only the bulk excitation can happen. However, there is no clear boundary between regions II and III. The DIIMFP related to surface excitation and Begrenzungs effect are restrained near to the surface and decay exponentially, $\exp \left( { - q_{\parallel } \left| z \right|} \right)$, with the increasing of the depth $z$^20^. In the present scenario, the depth-dependent DIIMFP can be written in the form^20^:

1 $$\sigma_{total} = \sigma_{bulk} + \sigma_{surf} + \sigma_{beg} ,$$

where

2 $$\sigma_{bulk} (z) = \frac{2}{{\pi v^{2} }}\int_{{q_{ - } }}^{{q_{ + } }} {dq\frac{1}{q}{\text{Im}} \left[ {\frac{ - 1}{{\varepsilon \left( {\vec{q},\omega } \right)}}} \right]} \;\Theta \left( { - z} \right);$$

3 $$\begin{gathered} \sigma_{surf} (z) = \frac{4\cos \alpha }{{\pi^{3} }}\int_{{q_{ - } }}^{{q_{ + } }} {dq\int_{0}^{{\frac{\pi }{2}}} {d\theta \int_{0}^{2\pi } {d\phi \frac{{q\sin^{2} \theta \exp \left( { - q_{\parallel } \left| z \right|} \right)}}{{\tilde{\omega }^{2} + q_{\parallel }^{2} v_{ \bot }^{2} }}} } } \times {\text{Im}} \left[ {\frac{ - 1}{{\varepsilon \left( {\vec{q}_{\parallel } ,\omega } \right) + 1}}} \right] \hfill \\ \times \left\{ {\left[ {2\cos \left( {\frac{{\tilde{\omega }z}}{v\cos \alpha }} \right) - \exp \left( { - q_{\parallel } \left| z \right|} \right)} \right] \times \left[ {\Theta \left( z \right)\Theta \left( {v_{ \bot } } \right) + \Theta \left( { - z} \right)\Theta \left( { - v_{ \bot } } \right)} \right] + \cos \left( { - q_{ \bot } \left| z \right|} \right)\left[ {\Theta \left( { - z} \right)\Theta \left( {v_{ \bot } } \right) + \Theta \left( z \right)\Theta \left( { - v_{ \bot } } \right)} \right]} \right\}; \hfill \\ \end{gathered}$$

4 $$\begin{gathered} \sigma_{beg} (z) = - \frac{2\cos \alpha }{{\pi^{3} }}\int_{{q_{ - } }}^{{q_{ + } }} {dq\int_{0}^{{\frac{\pi }{2}}} {d\theta \int_{0}^{2\pi } {d\phi \frac{{q\sin^{2} \theta \exp \left( {q_{\parallel } z} \right)}}{{\tilde{\omega }^{2} + q_{\parallel }^{2} v_{ \bot }^{2} }}{\text{Im}} \left[ {\frac{ - 1}{{\varepsilon \left( {\vec{q}_{\parallel } ,\omega } \right)}}} \right]} } } \hfill \\ \times \left\{ {\cos \left( {q_{ \bot } z} \right)\Theta \left( { - z} \right)\Theta \left( {v_{ \bot } } \right){ + }\left[ {2\cos \left( {\frac{{\tilde{\omega }z}}{v\cos \alpha }} \right) - \exp \left( {q_{\parallel } z} \right)} \right]\Theta \left( { - z} \right)\Theta \left( { - v_{ \bot } } \right)} \right\}. \hfill \\ \end{gathered}$$

In Eqs. (2)–(4), $\tilde{\omega } = \omega - qv\sin \theta \cos \phi \sin \alpha$, $q_{\parallel } = q\sin \theta$, $v_{ \bot } = v\cos \alpha$ and $E = {{v^{2} } \mathord{\left/ {\vphantom {{v^{2} } 2}} \right. \kern-\nulldelimiterspace} 2}$. $\alpha$ is defined as the angle between the surface normal and the electron moving direction. The upper and lower limits of the integrals are $q_{ \pm } = \sqrt {2E} \pm \sqrt {2(E - \omega )}$. So, according to this definition we have functional form of bulk and surface excitations and also for Begrenzungs term. Equation (2) defines the bulk excitation, which does not depend on the depth and represents the scattering of electrons inside a semi-infinite material. The Begrenzungs term (Eq. 4), occurring only inside the solid, indicates a decrease of the bulk inelastic cross section, which is due to the coupling between the volume and surface modes that are orthogonal^46^. Here we consider this effect separately instead of mixing with surface excitations. One may note that the Begrenzungs term gives negative values and obviously it is impossible to measure practically. The only way for detailed investigation of Begrenzungs effect is to perform a quantitative theoretical analysis based on the experimental spectra. The surface excitation occurs not only inside a solid but also above it in the vacuum near the surface (see Eq. 3). The momentum transfer-dependent ELF, ${\text{Im}} \left[ {{{ - 1} \mathord{\left/ {\vphantom {{ - 1} {\varepsilon \left( {q,\omega } \right)}}} \right. \kern-\nulldelimiterspace} {\varepsilon \left( {q,\omega } \right)}}} \right]$, in Eqs. (2)–(4) can be obtained by an extension from the long wavelength limit $q \to 0$, namely the optical ELF ${\text{Im}} \left[ {{{ - 1} \mathord{\left/ {\vphantom {{ - 1} {\varepsilon \left( \omega \right)}}} \right. \kern-\nulldelimiterspace} {\varepsilon \left( \omega \right)}}} \right]$, by assuming a dispersion relation. In this work, a FPA-Ritchie-Howie method^29^ is employed to extended the ELF, i.e. using the full Penn algorithm (FPA)^47^ to extend the ELF for the calculation of the bulk DIIMFP $\sigma_{bulk}$ while using Ritchie and Howie’s scheme^48^ for the calculation of the surface excitation DIIMFP $\sigma_{surf}$ and Begrenzungs effect term $\sigma_{beg}$.

Here we would like to highlight again that the Begrenzungs effect is a weakening effect on bulk excitation. So the Begrenzungs effect cannot exist alone, it is closely linked to bulk excitation. The inelastic scattering events are identified either as bulk or surface excitations. The measured or calculated electron spectra can be expressed as a sum of contributions of various scatterings in the form:

5 $$\begin{gathered} I_{total} \left( \omega \right) = \sum\limits_{{n_{b} = 0}}^{\infty } {\sum\limits_{{n_{s} = 0}}^{\infty } {I_{{n_{b} ,n_{s} }} \left( \omega \right)} } \hfill \\ = I_{{n_{b} { = 0},n_{s} { = 0}}} \left( \omega \right){ + }\sum\limits_{{n_{b} = 1}}^{\infty } {I_{{n_{b} ,n_{s} = 0}} \left( \omega \right)} + \sum\limits_{{n_{s} = 1}}^{\infty } {I_{{n_{b} = 0,n_{s} }} \left( \omega \right)} + \sum\limits_{{n_{s} = 1}}^{\infty } {\sum\limits_{{n_{s} = 1}}^{\infty } {I_{{n_{b} ,n_{s} }} \left( \omega \right)} } \;, \hfill \\ \end{gathered}$$

where $n_{b}$ and $n_{s}$ are the number of bulk and surface excitations, respectively. The first term in Eq. (5) represents the elastic peak, $I_{{0}} \left( \omega \right) = I_{{n_{b} { = 0},n_{s} { = 0}}} \left( \omega \right)$. The second term shows that the signal electrons suffer only bulk excitation. Due to the influence of Begrenzungs effect, this term can be expressed as $I_{bulk + beg} \left( \omega \right) = I_{beg} \left( \omega \right) + I_{bulk} \left( \omega \right)$. The third term is the contribution of electrons suffer only surface excitation, i.e. it represents the pure surface excitations, $I_{surf} \left( \omega \right)$. The last term contains the signal electrons which suffer both bulk and surface excitations in direct consequence of multiple scattering. We refer hereafter this term as the mixed term, $I_{mix} \left( \omega \right)$, as a superposition between bulk and surface excitations with Begrenzungs effect. So the Eq. (5) can be rewritten as:

6 $$\begin{gathered} I_{total} \left( \omega \right) - I_{0} \left( \omega \right) = I_{total}^{inel} \left( \omega \right) = I_{bulk + beg} \left( \omega \right) + I_{surf} \left( \omega \right) + I_{mix} \left( \omega \right) \hfill \\ = I_{bulk} \left( \omega \right) + I_{surf} \left( \omega \right) + I_{beg} \left( \omega \right) + I_{mix} \left( \omega \right). \hfill \\ \end{gathered}$$

Given an experimental REELS spectrum, the specific analysis steps of present method are: (a) extract the ELF from the experimental spectrum by the reverse MC method^12,24^; (b) perform a MC simulation of REELS spectrum by using the obtained ELF; (c) derive spectrum components as given in Eqs. (5), (6) from the MC simulated spectrum. One may also perform a quick analysis based on the existing ELF. In this work, REELS spectrum of Si at the primary energy of 5 keV is used as an example. Mott’s cross-section^49^ is used to describe electron elastic scattering in a MC simulation of REELS spectrum. The Thomas–Fermi–Dirac atomic potential^50^ is used in the calculation of Mott’s cross-section. We used ELF from^29^ below 200 eV, and Henke’s data^51^ for 200 eV–30 keV in the calculation of inelastic cross section. Although it has been reported that the negative DIIMFP in vacuum may indicate an energy gain of electrons due to the interaction with the surface plasmon^52^, however, its influence to the REELS spectra at the primary energy of 5 keV can be negligible. Therefore, in the present simulation of the electron spectra such energy gain has not been taken into account. The electrons suffer inelastic scatterings during transport in materials, which are identified either as bulk or surface excitations. The probability of surface excitation can be determined as $P_{surf} = {{\sigma_{surf} } \mathord{\left/ {\vphantom {{\sigma_{surf} } {\sigma_{total} }}} \right. \kern-\nulldelimiterspace} {\sigma_{total} }}$, which depends on the electron energy $E$, moving direction $\alpha$, depth $z$ and energy loss $\omega$. Hence, the specific type for each inelastic scattering can be determined by sampling. Using Eqs. (1)–(4), we can distinguish the type of inelastic scattering and count the number of bulk excitation or surface excitation in a MC simulated REELS spectrum. According to Eq. (6), three different components, i.e. $I_{bulk + beg} \left( \omega \right)$, $I_{surf} \left( \omega \right)$ and $I_{mix} \left( \omega \right)$, can be obtained.

In order to separate the bulk excitation component and Begrenzungs effect, a virtual situation was considered by assuming that the Begrenzungs effect does not exist. In this simulation the DIIMFP is as follows: $\sigma_{total} = \sigma_{bulk} + \sigma_{surf}$. Based on the results of the virtual simulation three different spectral components, i.e. $I_{bulk}^{{\prime }} \left( \omega \right)$, $I_{surf}^{{\prime }} \left( \omega \right)$ and $I_{mix}^{{\prime }} \left( \omega \right)$ are obtained. The Begrenzungs effect is a correction to the bulk excitation rather than to play a major role in the evaluation. We can assume that, there is no difference between $I_{bulk} \left( \omega \right)$ and $I_{bulk}^{{\prime }} \left( \omega \right)$, so Begrenzungs effect component can be written as $I_{beg} \left( \omega \right) = I_{bulk + beg} \left( \omega \right) - I_{bulk}^{{\prime }} \left( \omega \right) = I_{bulk + beg} \left( \omega \right) - I_{bulk} \left( \omega \right)$.

## Data Availability

The datasets generated during and/or analyzed during the current study are available from the corresponding author on reasonable request.
